# Trends in the Incidence and Severity of Injuries Sustained by Riders of Electric Bikes and Powered Scooters: A Retrospective Cross-Sectional Study

**DOI:** 10.3390/medicina58070934

**Published:** 2022-07-14

**Authors:** Bahaa Haj Yahya, Helena Demetriou, Adi Zelnik, Nir Cohen, Michael J. Drescher, Gavriel Chaushu, Yafit Hamzani

**Affiliations:** 1 Oral and Maxillofacial Private Clinic, Herzliya 4672211, Israel; bahaa.hag@gmail.com; 2 Rabin Medical Center, Department of Oral and Maxillofacial Surgery, Beilinson Hospital, Petach Tikva 4941492, Israel; drlilidemetriou@gmail.com (H.D.); gabi.chaushu@gmail.com (G.C.); 3 Maccabi-Dent, Holon 4941492, Israel; adi.zelnik@gmail.com; 4 Rabin Medical Center, Department of Orthopedic Surgery, Beilinson Hospital, Petach Tikva 4941492, Israel; nirco2@clalit.org.il; 5 Sackler Faculty of Medicine, Tel Aviv University, Tel Aviv 6997801, Israel; michaeldr@clalit.org.il; 6 Rabin Medical Center, Department of Emergency Medicine, Beilinson Hospital, Petach Tikva 4941492, Israel; 7 The Maurice and Gabriela Goldschleger School of Dental Medicine, Tel Aviv University, Tel Aviv 6997801, Israel

**Keywords:** emergency department, age, electric bikes, powered scooters, injury

## Abstract

*Background and Objectives:* The worldwide increase in electric bike (E-bike) and powered scooter (P-scooter) use in recent years has been accompanied by an increase in associated injuries to riders. The aim of this study was to evaluate trends in the incidence and types of E-bikes and P-scooter-related injuries in riders evacuated to a tertiary ED. *Materials and Methods:* A retrospective cross-sectional design was used. The cohort included 1234 patients referred to the emergency department (ED) of a tertiary medical center in 2014–2020 for injuries sustained while riding an E-bike or P-scooter. Demographic, clinical, and injury data were collected from the medical files, and injury rates were evaluated over time. *Results*: The results showed that the annual number of ED visits by injured E-bike and P-scooter riders increased steadily over the study period concomitant with an increase in ED referrals for hospitalization, indicating severe injury. The upper and lower extremities were the most frequent anatomic sites of injury in every year of the study, with variations among the different age groups. *Conclusions:* Our findings suggest a need for safety regulations for riders who operate two-wheel powered vehicles, such as licensing requirements and mandatory protective gear, especially for anatomic sites most at risk.

## 1. Introduction

Electric bicycles (E-bikes) and powered scooters (P-scooters) have become a progressively popular mode of transportation worldwide [1,2,3,4], and their growing use has been accompanied by an increase in injuries to riders [5,6]. Between 2000 and 2017, 133,872 injuries associated with E-bikes and P-scooters were registered by the National Electronic Injury Surveillance System in the United States [1]. From 2013 to 2017, there was a precipitous increase in the number and severity of injuries of E-bike and P-scooter riders who presented at emergency departments (EDs) [1]. Compared with injuries involving pedal-operated bicycles, E-bike-related injuries were more often characterized as “internal” and were more likely to lead to hospitalization [1].

The aim of the present study was to evaluate the incidence of injuries to E-bike and P-scooter riders evacuated to a tertiary ED over a 6-year period, with a focus on severe injuries warranting hospital admission.

## 2. Materials and Methods

A retrospective, cross-sectional study was conducted in the ED of a tertiary medical center in Israel from January 2014 to March 2020. Primary search of the healthcare database using the keywords “electric scooter” or/and “electric bike” and/or “powered scooter” or/and “powered bike” and “injury/ injured” yielded a total of 1417 patients. On further screening, 1234 patients were found to have been riders involved in an E-bike or P-scooter accident for whom all relevant data were available. Their medical records were reviewed for demographics (age, gender), type of electric vehicle used (E-bike, P-scooter), hospitalization (yes/no), length of hospitalization (if relevant), and anatomic site of injury. Trends in the incidence and severity of injuries over time and by age were evaluated. The study protocol was approved by the Helsinki Committee of Rabin Medical Center (approval number 0194-20-RMC). 

The data were analyzed using SPSS, version 25 (IBM^®^, Armonk, NY, USA). Continuous variables are described by mean and standard deviation, and discrete variables by frequency. 

## 3. Results

### 3.1. Patient Characteristics

The cohort was comprised of 1234 patients, 934 male (75.7%) and 300 female (24.3%), of mean age 31.52 ± 14.77 years (median, 28 years). Ninety patients (7.3%) required hospitalization, for a mean duration of 5.44 ± 0.12 days. Three patients (0.2%) died before arriving to the hospital or at initial ED treatment, and none of them were hospitalized prior to their deaths. E-bikes were involved in 79.5% of accidents and P-scooters in 20.5%. Accidents occurred with helmets 9.6% of the time.

The complete descriptive statistics of the cohort are shown in Table 1.

### 3.2. Trends in Incidence and Types and Severity of Injuries and Effect of Age

**Figure 1:**
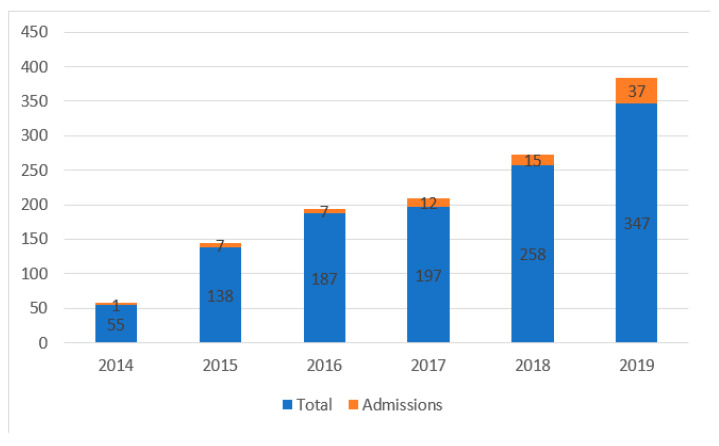
Number of admissions from total cohort by year.

**Figure 2:**
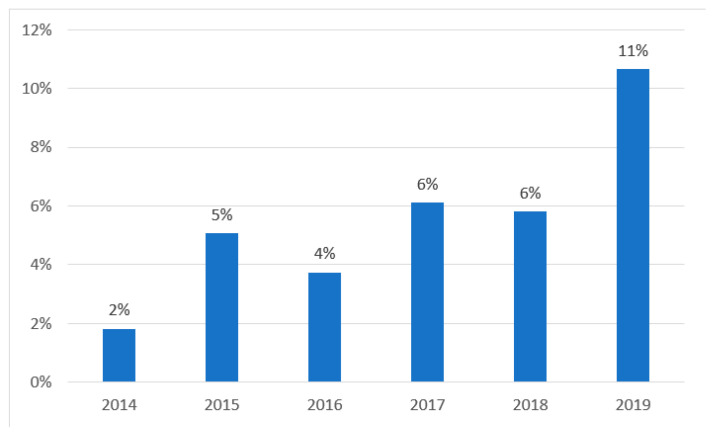
Percentage of admissions from total cohort by year.

The number of patients who visited the ED because of an E-bike or P-scooter injury increased in each consecutive year over the study period, from 2014 to 2019, as did the number of injured riders referred for hospitalization. For example, during the last 2 years evaluated, the number of riders evacuated to the ED rose from 247 in 2018 to 348 in 2019, among whom 6% and 11%, respectively, were hospitalized. Figure 1 depicts the increase in incidence of injuries, and Figure 2, the increase in severity of injuries. (Values for 2020 were not included in this analysis because the study was terminated in March of that year).

Analysis by age revealed that patients aged 30 years or less accounted for 56% of the cohort (694/1234), but only 38% of those were referred for hospitalization (34/90). Corresponding rates for patients older than 30 years were 44% and 62%, respectively.

Overall, the upper and lower limbs were the most frequent body parts injured, in 689 (55.8%) and 609 (49.4%) patients, respectively. Analysis over time showed that they were the most frequent anatomic sites of injury in every year of the study, from 2014 to 2020 (Table 2).

The difference in frequency of injury was about 20% higher for the extremities than for other body parts (Table 2). The next most-common sites of injury were the face, in 268 patients (21.7%) and the head, in 209 (16.9%).

When the cohort was divided by age (in decades), the extremities remained a frequent site of injury (Table 3), although there were variations among the groups.

For example, in the oldest patients (age 90–99 years), the head and face accounted for 50% and 25% of cases, respectively. The results for sites of injury by age are depicted in Table 3.

## 4. Discussion

The present investigation of trends in the incidence and severity of injuries to E-bike and P-scooter riders presenting to a tertiary ED showed that the number of ED visits increased between each year of the study to the next over the 6-year study period. There was a parallel increase over time in the severity of injuries sustained by the riders, represented by the number of referrals for hospital admission from the ED. These results support earlier studies of E-bike and P-scooter accidents worldwide [1,4,6,7,8].

Our results are also in line with population studies on patterns of ED use and the effect of high case load. A report based on data derived from the National Emergency Department Sample in the United States showed that between 2010 and 2014 [9], the estimated total number of ED visits increased from 128.9 million (95% CI 123.6–134.4) to 137.8 million (95% CI 131.1–144.5), with a corresponding increase in ED visit rates from 2.97% to 6.70%. ED visit rates per 1000 persons rose significantly, from 16.9 to 432.5 (*p* = 0.0136). The rate of growth in ED visits (1.7%) was more than twice that of the general population (0.7%) [9]. Using a cumulative logit model to analyze changes in severity of injuries seen in the ED over time, others predicted that as ED occupancy rose from 25 to 75 patients, the probability of a patient being triaged as high acuity increased by about 50%, and the probability of a patient being categorized as requiring hospital admission increased by around 25% [5]. The odds of being hospitalized versus discharged from the ED increased by 1.007-fold for each additional patient in the ED at the time of the disposition decision. The authors concluded that ED crowding may be associated with a higher rates of case classification as higher acuity and of patient referral for hospitalization [5]. These findings may explain the concomitant increase in our study of total injuries and hospitalization for severe injuries in E-bike and P-scooter riders.

In terms of rider age, our analysis showed that although patients less than 30 years old accounted for the majority of the cohort (56%), patients who were older than 30 years were more likely to be hospitalized (62%). The higher probability of more severe injury in older riders conforms with the results of a recent multicenter study from Vienna which reported an increase in Injury Severity Score with an increase in rider age [10]. Accordingly, DiMaggio et al. [1], in a study extending from 2000 to 2017, found that E-bike injuries in the US have been increasing dramatically since 2013 particularly among older persons.

In the present study, the upper and lower extremities were the most frequent anatomic sites of injury, in 55.8% and 49.4% of the cohort, respectively, followed by the face (21.7%) and head (16.9%). Similarly, retrospective observational studies based on imaging scans reported that most P-scooter injuries were categorized as high-energy trauma and affected primarily the head and upper extremities [10], specifically the face, wrist, hand, and elbow [11]. Accordingly, most imaging scans performed in a retrospective cohort of P-scooter riders in Southern California were for injuries to the distal upper extremities (36.5%), head (29.7%), and distal lower extremities (20.1%) [12], and an analysis conducted in a tertiary trauma center in Australia following the introduction of a scooter-share scheme found that injuries usually involved the upper limbs and head [13]. The extremities and face were also reported as the most frequent sites of injury in studies of different types of two-wheel electric vehicles [6,7,8].

The annual increase in the number of ED visits associated with electric-vehicle-related injuries affects not only patients but the entire medical system. A study conducted in New Zealand found that there were no cases of P-scooter-related injuries in 2018, whereas on every day during 2019, on average, one ED bed was occupied by a patient injured in a P-scooter accident for 2 h and 44 min [7].

On the basis of our results and the medical literature, we suggest that more stringent control policies for P-scooter and E-bike use are needed to decrease the incidence of related injuries and rates of hospital admission for these injuries. Larger worldwide multicenter studies may help to assess global trends in electric vehicle injuries.

## 5. Conclusions

Despite the widespread use of E-bikes and P-scooters, awareness of the incidence, severity, and repercussions of related injuries remains low. The present study showed a spiraling increase in the incidence and severity of injuries sustained by E-bike and P-scooter riders in recent years. These findings also have implications for the healthcare system itself in terms of the contribution of two-wheel electric vehicle injury to ED crowding and the work volume of medical teams. This study may provide some guidance towards the formulation of effective safety legislation, such as licensing of riders of two-wheel electric vehicles, and national educational campaigns to promote the use of protective gear, especially for the head and extremities.

## Figures and Tables

**Table 1:** Demographic and clinical characteristics of the cohort (N = 1234).

Characteristics	Value
Demographics	
Male/Female	934 (75.7%)/300 (24.3%)
Age (yr), mean ± SD, range	31.52 ± 14.77, 5–94
Vehicle type	
E-bike	980 (79.5%)
P-scooter	253 (20.5%)
Site of injury	
Upper limbs	689 (55.83%)
Lower limbs	609 (49.35%)
Face	268 (21.72%)
Head	209 (16.94%)
Stomach and pelvis	118 (9.56%)
Back	102 (8.27%)
Chest	100 (8.10%)
Neck	31 (2.51%)
None	4 (0.32%)

Values are n (%) unless otherwise indicated.

**Table 2:** Anatomic site of injury by year.

Injury Site	2014	2015	2016	2017	2018	2019
Upper limbs	32 (36.78%)	65 (27.54%)	95 (28.61%)	120 (33.80%)	150 (33.11%)	192 (32.54%)
Lower limbs	31 (35.63%)	75 (31.78%)	92 (27.71%)	103 (29.01%)	133 (29.36%)	151 (25.59%)
Face	7 (8.05%)	32 (13.56%)	39 (11.75%)	40 (11.27%)	49 (10.82%)	93 (15.76%)
Head	8 (9.10%)	23 (9.75%)	38 (11.45%)	30 (8.45%)	43 (9.49%)	61 (10.34%)
Stomach and pelvis	3 (3.45%)	13 (5.51%)	25 (7.53%)	15 (4.23%)	27 (5.96%)	36 (6.10%)
Back	2 (2.30%)	14 (5.93%)	23 (6.93%)	18 (5.07%)	22 (4.86%)	23 (3.90%)
Chest	2 (2.30%)	12 (5.08%)	15 (4.52%)	21 (5.92%)	20 (4.42%)	27 (4.58%)
Neck	2 (2.30%)	2 (0.85%)	3 (0.90%)	8 (2.25%)	7 (1.59%)	7 (1.19%)
None	0	0	2 (0.60%)	0	2 (0.44%)	0

**Table 3:** Anatomic site of injury by age group.

Injury Site	<18 yr	18–29 yr	30–39 yr	40–49 yr	50–59 yr	60–69 yr	70–79 yr	80–89 yr	90–99 yr
Upper limbs	84 (38.18%)	299 (32.12%)	159 (31.55%)	69 (29.74%)	31 (26.27%)	26 (39.39%)	18 (33.96%)	3 (33.33%)	0
Lower limbs	86 (39.09%)	259 (27.82%)	142 (28.17%)	60 (25.86%)	33 (27.97%)	12 (18.18%)	16 (30.19%)	2 (22.22%)	1 (25.00%)
Face	10 (4.55%)	123 (13.21%)	72 (14.29%)	35 (15.09%)	13 (11.02%)	8 (12.12%)	5 (9.43%)	1 (11.11%)	1 (25.00%)
Head	10 (4.55%)	90 (9.67%)	48 (9.52%)	25 (10.87%)	16 (13.56%)	14 (21.21%)	5 (9.43%)	1 (11.11%	2 (50.00%)
Stomach & pelvis	11 (5.00%)	55 (5.91%)	25 (4.96%)	14 (6.03%)	8 (6.78%)	1 (1.52%)	4 (7.55%)	1 (11.11%)	0
Back	12 (5.45%)	50 (5.37%)	26 (5.16%)	6 (2.59%)	5 (4.24%)	1 (1.52%)	2 (3.77%)	1 (11.11%)	0
Chest	6 (2.73%)	42 (4.51%)	23 (4.56%)	16 (6.90%)	10 (8.47%)	2 (3.03%)	1 (1.89%)	0	0
Neck	1 (0.45%)	10 (1.07%)	9 (1.79%)	7 (3.02%)	2 (1.69%)	1 (1.52%)	2 (3.77%)	0	0
None	0	3 (0.32%)	0	0	0	1 (1.53%)	2 (3.77%)	0	0

## Data Availability

The authors confirm that the data supporting the findings of this study are available within the article.
